# Microbial Community, Metabolic Potential and Seasonality of Endosphere Microbiota Associated with Leaves of the Bioenergy Tree *Paulownia elongata* × *fortunei*

**DOI:** 10.3390/ijms23168978

**Published:** 2022-08-11

**Authors:** Małgorzata Woźniak, Anna Gałązka, Anna Marzec-Grządziel, Magdalena Frąc

**Affiliations:** 1 Department of Agricultural Microbiology, Institute of Soil Science and Plant Cultivation—State Research Institute, Czartoryskich 8, 24-100 Pulawy, Poland; 2 Institute of Agrophysics, Polish Academy of Sciences, Doswiadczalna 4, 20-290 Lublin, Poland

**Keywords:** *Paulownia*, endomicrobiome, structural and functional diversity, bacteria, next-generation sequencing

## Abstract

The microbial structure and metabolic function of plant-associated endophytes play a key role in the ecology of various environments, including trees. Here, the structure and functional profiles of the endophytic bacterial community, associated with *Paulownia elongata* × *fortunei*, in correlation with seasonality, were evaluated using Biolog EcoPlates. Biolog EcoPlates was used to analyse the functional diversity of the microbiome. The total communities of leaf endophyte communities were investigated using 16S rRNA V5–V7 region amplicon deep sequencing via Illumina MiSeq. Community level physiological profiling (CLPP) analysis by the Biolog EcoPlate™ assay revealed that the carboxylic acids (19.67–36.18%) and amino acids (23.95–35.66%) were preferred by all by all communities, whereas amines and amides (0.38–9.46%) were least used. Seasonal differences in substrate use were also found. Based on the sequencing data, mainly phyla Proteobacteria (18.4–97.1%) and Actinobacteria (2.29–78.7%) were identified. A core microbiome could be found in leaf-associated endophytic communities in trees growing in different locations. This work demonstrates the application of Biolog EcoPlates in studies of the functional diversity of microbial communities in a niche other than soil and shows how it can be applied to the functional analyses of endomicrobiomes. This research can contribute to the popularisation of Biolog EcoPlates for the functional analysis of the endomicrobiome. This study confirms that the analysis of the structure and function of the plant endophytic microbiome plays a key role in the health control and the development of management strategies on bioenergy tree plantations.

## 1. Introduction

All living organisms, including plants, are colonised by a diverse and complex array of microorganisms, encompassing bacteria, fungi, archaea, and protozoa. These associations between the host plant and its related microbiota are regarded as a close-knit unit known as the “holobiont”. The information about the microorganisms and their genomes inhabiting a host plant is defined as the host microbiome. The “plant holobiont” concept implies that evolutionary selection takes place between the host and unique microbiomes as well as within microbe–microbe members [1,2]. Plant microbiota live in different organs and tissues, including leaves, roots, stems, seeds, fruits, bulbs and flowers [3,4], and previous studies have indicated that the microbiota diversity is a feature forming part of the extended phenotype of the host plant [5,6]. Previous reports on the structure and function of microorganisms associated with plants have focused on the roots and rhizosphere microbiome [7,8]. Later studies discovered that the phyllosphere also offers habitats to complex, although less different, microbiota [9]. The phyllosphere represents a fluctuating, unstable and relatively harsh environment. Microbial communities associated with the phyllosphere are exposed to various environmental factors, i.a., ultraviolet radiations, temperature and humidity fluctuations, with negative impacts on the growth of most microorganisms [10]. Nevertheless, this environment comprises a living habitat for lots of various communities of microorganisms well adapted to these unfavourable conditions [11,12]. The leaves are colonised by diverse microbes, some of which inhabit the surface of plant organs (epiphytes), whereas others inhabit the interior of plant organs (endophytes) [12,13,14]. All these microorganisms exhibit different ecological interactions with their host plants from beneficial and commensal symbioses to pathogenic relationships [14,15]. Among the beneficial interactions between the plant and endophyte, direct or indirect mechanisms, such as increasing nutrient acquisition, improving resilience to abiotic stresses, and resistance to pathogens, can be mentioned. Plant-dwelling endophytes can also detoxify environmental pollutants and directly stimulate plant growth through the synthesis of phytohormones [6,16,17,18,19]. Furthermore, leaf-associated microorganisms can impact the exchange of gases and plant-derived volatiles [20] and modify patterns of herbivory [21]. Previous studies show that endophytic microbiota are characterised by the production of unique secondary metabolites that are useful in biotechnology, agriculture and pharmacy [22,23,24].

Many studies have shown that microbial communities show a great diversity of metabolic capabilities, which have emerged as a key determinant of various aspects of the host plant biology [22,23,24]. The microbiome influences the physiological, reproductive and developmental activities, and can shape phenotypes of the host [6,25,26,27]. The structure of the phyllosphere microbial communities is not random; its formation and development are continuous and dynamic process. The determinants of the taxonomic composition, diversity and the potential functionality of microbiome include the host species, tissue type, growth stage, varieties, biogeography, soil type, atmospheric conditions, cropping history, etc. [9,13,28,29,30]. 

Climate change causes radical changes, such as global warming, increasing atmospheric carbon dioxide concentration, loss of biodiversity, altered natural ecosystems and ecosystem services and species extinction, which are currently some of the main challenges for society and the world. Various strategies are currently being promoted to switch from the use of fossil fuels to renewable energy. Plantations of trees with short rotations are a promising tool for decreasing the concentration of atmospheric CO_2_, but were first introduced to produce biomass for energy [31,32,33,34]. *Paulownia* spp. are trees that belong to the Paulowniaceae family. *Paulownia* is also known by many other names, including Princess Tree, biotree, Royal tree and Phoenix tree. *Paulownia* is a deciduous tree species, able to reach very high growth rates under favourable conditions. It is well adapted to growing and functioning in a wide range of changing soil and climatic conditions. *Paulownia* is one of the few trees in which the C_4_ photosynthesis pathway occurs. The leaves of young trees are large, reaching a width of up to 80 cm and contain large amounts of proteins (approximately 20%), fats, sugars, nitrogen, phosphorus, calcium, potassium, zinc and iron [31,32,35,36,37]. According to Popova and Baykov 2013 [38], the leaves of *Paulownia* can be used as an alternative feed and green manure.

Recently, interest in the leaf microbiome has increased. Analysis of the structure and function of the plant endophytic microbiome is a critical component that enables an understanding of microbial ecology, host plant biology, the positive and negative impacts of microbes on plants and the role of the microbiome as a reservoir of additional genes and functions. The in-depth exploration of the structure and functioning of endophytic microbes may constitute the basis to develop holistic management strategies aiming to deal with the common biotic and abiotic factors affecting tree crops. By using metagenomics methods and physiological profile analyses, it is possible to expand our knowledge of this interesting group of both cultivable and non-cultivable microbial communities [39,40].

The Biolog EcoPlates test is widely used in the analysis of the microbiome and is represented by community-level physiological profiles (CLPP). The EcoPlate test is used for the community-level physiological profiling (CLPP) of microorganisms, contains 31 of the most useful carbon sources from, altogether, five compound groups—carbohydrates, carboxylic acids, amines and amides, amino acids and polymers—as well as a blank well as a control, all these replicated thrice [41,42,43]. Ros et al. (2008) [44] argue that the Biolog test does not reflect the functional capacities of all the microorganisms in the sample, but only reflects the capacities of a limited subset of the microbial community. However, physiological profiling (CLPP) is an appropriate technique to compare microbial functional diversity over space and time to changes in the environment [41,42,43]. 

To date, numerous studies have been conducted to reveal the functions, diversity and community structure of leaf-associated endophytes [45,46,47], yet there are still knowledge gaps that need to be addressed. Most studies have focused on other taxa, such as *Populus* spp., *Salix* spp. and *Eucalyptus* spp. [48,49,50], whereas information about the bacterial communities of leaves in *Paulownia* plantations is limited [32]. In this context, this study investigates the *Paulownia*-leaves endosphere microbiome, including structure, diversity and metabolic function. Moreover, a very important aspect in this research are the spatio–temporal variations. Insight into the composition, dynamics and functions of the interaction between endophytic bacterial microbiota and *Paulownia*-leaf will shed light on *Paulownia* microbial resources, which may drive the adaptation of terrestrial ecosystems to global changes (yeasts/fungi were not selected against in this preparation, but they would not be expected to dominate community activity in these assays, which better suit bacteria). In addition, such resources can also be used in biotechnological application.

The overall aim of our research is to evaluate the microbial community composition and metabolic potential of endosphere microbiota associated with leaves of the bioenergy tree—*Paulownia elongata* × *fortunei*. 

## 2. Results

### 2.1. CLPP Analysis Using Biolog EcoPlates 

Examination of the functional diversity and evaluation of the physiological profile of microbial communities in samples collected from Paulownia leaves were performed by phenotype microarray analysis based on the use patterns of 31 single carbon sources. For the best differentiation of bacterial communities, it is important to select the appropriate incubation time at which the average colour development (AWCD) of all carbon sources reaches a peak value before the constant phase. In addition, the selection of an appropriate bacterial inoculum concentration is crucial. In this study, the dilution 10^2^ contained numerous bacteria and AWCD reached a peak after only 48 h, whereas the dilution 10^4^ likely had a relatively low number of bacteria. Therefore, the dilution of 10^3^ was chosen for measuring the metabolic activity of the microbial communities based on the AWCD values. The time selected for analysis was 168 h. Endophyte communities within this period showed optimal capability to use the carbon substrates in the Biolog EcoPlates. 

The AWCD indices of all carbon substrates in Paulownia leaves fluctuated within 0.414–1.215 (Table 1). Overall, the samples II_PA and II_GA showed the highest AWCD values, followed by II_BA and I_GuS. This indicates that the microbial communities in these samples were characterised by the most intense metabolic activity. In contrast, the lowest AWCD was observed for the I_PS and I_GS samples. The metabolic diversity of carbon source use in Paulownia leaves by microbial communities after 168 h of incubation using a set of substrates on Biolog EcoPlates was reflected by functional diversity indices. The Shannon diversity index (H′), Shannon evenness index (E), and Richness index (R) of Paulownia microbial communities are shown in Table 1. The highest mean Shannon diversity (H) index values were detected for leaf microbiota in autumn from Granice, followed by samples II_B, P and II_O, whereas the lowest values were found for the I_GS sample. Similarly, sample II_GA had a significantly higher (*p*  <  0.05) value of the richness index (R) than the other samples (Table 1). A similar trend in the functional diversity of leaf microbiota was observed between the two sampling periods. Overall, the metabolic and functional diversity indices values of AWCD, H′, R and E of the Paulownia-leaf microbiota from the different localisations were higher in autumn than in summer. 

**Figure 1:**
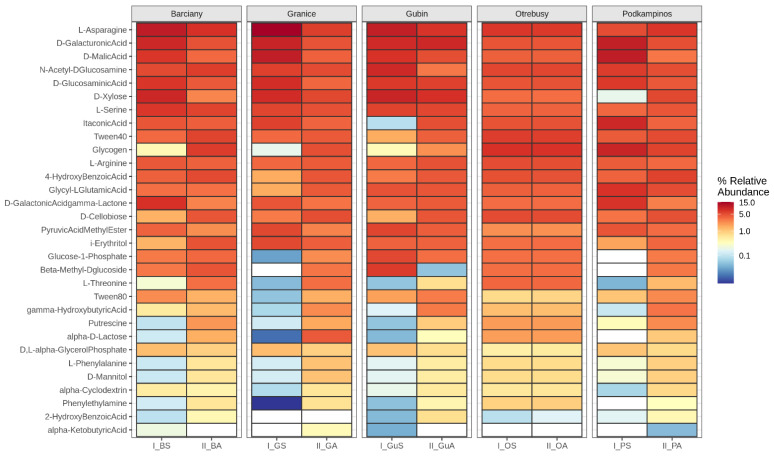
Heat map of the carbon sources utilisation patterns based on the average absorbance values after 168 h of incubation.

To illustrate the Biolog results and metabolic profiles, the use pattern of the 31 carbon substrates is presented as a heatmap graph (Figure 1). The highest functional richness (positive wells) among the studied endophytes was observed for the leaves sampled from Granice in autumn season. The microbial communities could metabolise 27.33 of the 31 carbon sources (Table 1), with the highest use rates for L-asparagine, followed by D-xylose, N-acetyl-D′glucosamine and D-cellobiose (Figure 1). The lowest functional richness was detected in the I_G and I_B samples during summer, respectively collected from Granice and Barciany; the microbial communities were able to use only 12.33 and 13.00 substrates, respectively (Table 1). Generally, the microbial communities in all leaf samples showed a similar trend in their use of the individual carbon sources. This is mainly seen in the low metabolising activity for compounds such as tween 40, gamma-hydroxybutyric acid, L-threonine and others in samples collected during the summer period. Notably, L-asparagine, D-galacturonic acid and N-acetyl-D glucosamine were the most intensively metabolised by microorganisms in all samples, regardless of the sampling season (Figure 1). In contrast, alpha-ketobutyric acid and 2-hydroxy benzoic acid were the least used (Figure 1).

**Figure 2:**
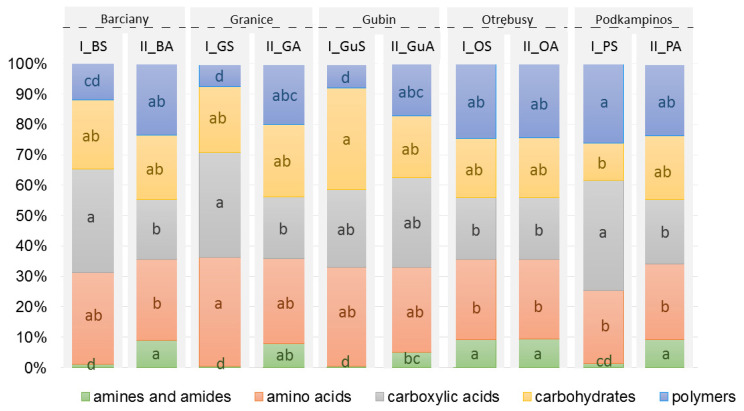
Relative utilisation of categorised carbon sources: amines and amides, amino acids, carboxylic and acetic acids, carbohydrates and polymers. after 168 h of plate incubation. Different letters indicate significant differences (*p* < 0.05, *n* = 3) by Tukey’s HSD test.

The 31 different substrates on the Biolog EcoPlates were assigned into five categories: carboxylic acids, carbohydrates, amino acids, polymers and amines/amides. The relative use of the substrate groups is presented in Figure 2. The utilisation of carbon sources differed among the leaf samples. Overall, the microbial communities in samples collected in both periods mostly used carboxylic acids and amino acids. Moreover, the higher degree of carboxylic acids and amino acids use was more closely associated with the leaf microbiota in summer than in autumn. In contrast, the lowest utilisation of carbon substrates was found for amines/amides, followed by polymers. These two groups were more significantly used in summer (Figure 2). The utilisation of all categorised substrates by the microbial communities differed significantly (*p* < 0.05). The greatest variation was observed for carboxylic acids and amines, where five different homogeneous groups were recorded using Tukey’s HSD test.

### 2.2. Structural Analysis of the Microbial Community

#### Bacterial Community Composition 

**Figure 3:**
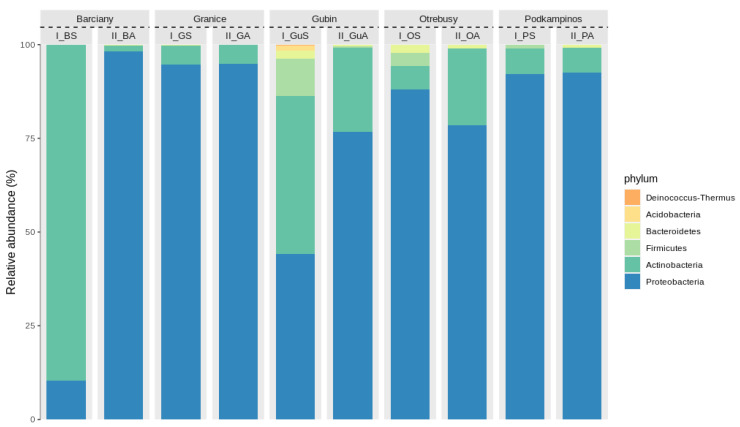
Histogram showing the relative abundance of the TOP six bacterial communities at the phylum level.

In this study, the bacterial communities on the leaves of Paulownia samples from different localities and sampled in different seasons were analysed by 16S rDNA amplicon sequencing. The relative abundances of the six most abundant phyla are shown in Figure 3. The results revealed that the group of bacteria in the leaves samples at both seasons were mainly Proteobacteria (from 18.4% to 97.1%), except the leaf sample collected from Barciany. Notably, there were differences between the relative abundance of certain bacterial phyla in the two sampling periods. Among the samples collected during the summer, the sample from Barciany showed a different trend regarding microbial taxonomic analysis at the phylum level. Actinobacteria (78.7%) was dominant in this sample (I_B), and Proteobacteria was the second most abundant type. During the autumn period, a significant decrease in the occurrence of these bacteria (Actinobacteria) was observed, from 78.7% to 2.29%. On the other hand, an increase in bacteria classified as Proteobacteria was observed, from 18.44% to 97.19%. Similar results were observed for leaf samples from Gubin, albeit with smaller differences in the relative abundance of Actinobacteria and Proteobacteria. 

**Figure 4:**
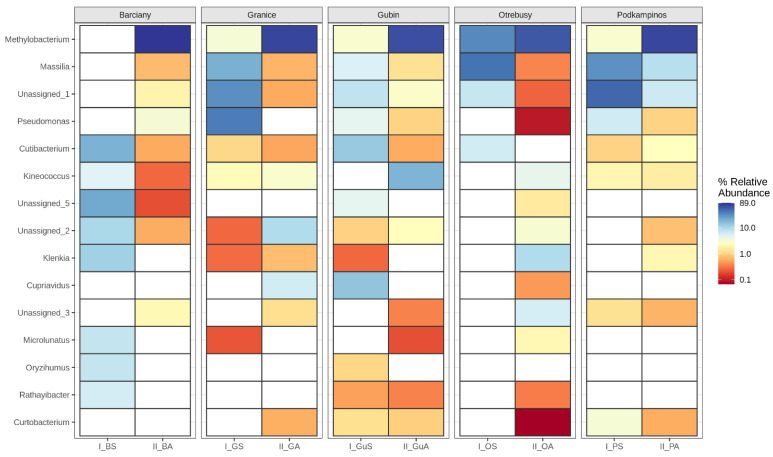
Heatmap showing the relative abundances of bacterial communities at the genus level.

Heatmap analysis of the relative abundances of the most abundant genera showed clear differences in the bacterial community structures among the samples from the five localities (Figure 4). Sequences assigned to *Methylobacterium* were most abundant in all communities during autumn, accounting for 60.2–88.4% of the total high-quality sequences. *Massilia* was the dominant genus (>5% relative abundance) in I-GS, II_GuA, I_O, I_P and II_P samples. *Pseudomonas* was the most abundant genus (38.4% relative abundance) in leaves collected from Gubin during summer, whereas *Cuprivadus* was characteristic of the leaf microbiomes of the II_GA and I_GuS sample. In summary, the distributions of the genera differed greatly, independent of the localisation as well as the season of collected samples.

**Figure 5:**
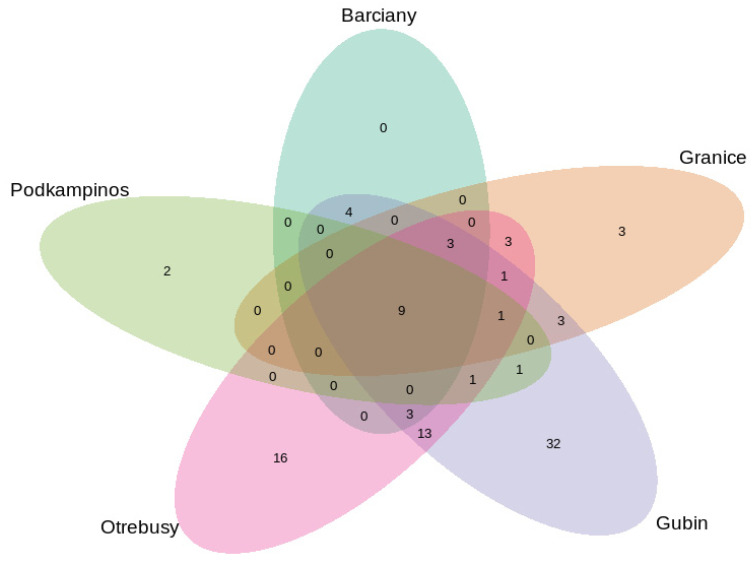
Venn diagram showing the numbers of shared and unique bacterial genera in leaves from from different localities.

Using Venn diagrams, information on the unique and shared bacterial genera among different localities was obtained (Figure 5). The composition of the core endomicrobiome of leaves from *Paulownia* consisted of nine bacterial genera, namely *Methylobacterium*, *Klenkia*, *Pseudomonas*, *Massilia*, *Cutibacterium*, *Kineococcus*, Unassigned_1, Unassigned_2 and Unassigned_3. The largest number of unique genera (32) was found for the samples from Gubin, sharing the most bacterial genera with the samples from Otrebusy (13). The leaf microbiomes from Otrebusy had 16 unique bacterial genera, whereas those from Granice had 3 unique bacterial genera, of which 3 were shared with the samples from Gubin. In contrast, the bacterial communities of the leaves from Barciany had no unique bacterial genera. 

## 3. Discussion

Various microorganisms associated with plants have a significant effect on plant fitness, health and nutrition status. Studies on endophytic bacterial community are most restricted to crop plants, whereas in tree species, such studies are limited to reports from forest ecosystems [50]. *Paulownia* sp. is one of the most important industrial trees, cultivated worldwide for multiple aims, including the production of biomass and feedstock for bioenergy. Moreover, *Paulownia* plantations have a large potential to sequestration of atmospheric CO_2_ [31,32,35,37]. The microbiome associated with plants is a highly complex network regarding structure and functional diversity, as well as spatial and temporal organisation. To obtain a better understanding of microbial functioning and taxonomy, the endophytic bacteria on the leaves of *Paulownia* sp. were investigated by the Biolog EcoPlates method and high-throughput sequencing. The aim of this study was to determine the community composition of endophytic bacteria from *Paulownia* clones growing under field conditions. Only little is known about the endophytic bacterial community harbored by *Paulownia* plants from other studies.

### 3.1. Community-Level Physiological Profiling

The Biolog EcoPlate test enables the characterisation of the diversity of community-level physiological profiles (CLPP) [51]. It is a simple method based on the metabolic properties of microorganisms inhabiting various ecological niches, for example, soil [52], freshwater [53], sediments [54], wastewater [55] and sewage sludge [51]. However, only a few studies have investigated the microbial functions of microorganisms by Biolog EcoPlates in the plants [32,56,57]. The results of the Biolog EcoPlate analysis reflect the metabolic activity and potential functional diversity of groups of microorganisms exposed to various factors, e.g., environmental stress. Microorganisms react quickly to any disturbances and environmental stresses, and changes in their metabolic profile may be a good and early indicator of changes in the ecosystem [58,59]. 

The results of this study showed that the metabolic activity of the *Paulownia* leaf microbiome based on carbon sources utilisation increased with the incubation time, which is consistent with the previous finding [32]. In the presented experiment, low values of the AWCD index, describing the total metabolic activity of the microbial community in a given sample, were observed. AWCD values range from 0.414 to 1.179. The microbial populations of *Paulownia* leaves exhibited a relatively low metabolic activity. Similarly, Szymańska et al. 2013 [40], Banik et al. 2017 [57] and Dresler et al. 2021 [60] noted low AWCD values for microbes from leaves of different plant species. Moreover, Szymańska et al. 2013 [40] emphasised that the total metabolic activity based on the AWCD index was lower than that of the rhizosphere and soil microorganisms. In our previous study [52], we assessed the microbial and biochemical properties of two soils with different textures in agroforestry plantations of *Paulownia elongata* × *Paulownia fortunei*, with regard to the analysis of their potential for the reclamation and redevelopment of abandoned lands. In this research, the AWCD index as an indicator of general microbial activity, ranged from 1.48 to 2.23. Most likely, this is related to the fact that the phyllosphere, including leaves, represents a fluctuating, unstable and relatively harsh environment. Microbial communities associated with the phyllosphere are exposed to various environmental factors, such as ultraviolet radiations, temperature, and humidity fluctuations, constricting the growth and activity of most microorganisms [10]. 

Analysis of the preferences of microorganisms for certain carbon sources revealed that carboxylic acids and amino acids were preferred, whereas amines and amides were the least metabolised. In a previous study on the structural and functional diversity of microorganisms inhabiting *Paulownia* leaves [32], we found that all microbial communities used carbohydrates abundantly, and in contrast, amines and amides were used the least. Similar results were obtained by Dresler et al. 2021 [60], who evaluated the functional diversity of endobacteria from leaves of *Cucumis sativus* using the Biolog EcoPlate. Banik et al. 2017 [57] determined the metabolic profiles of the epiphytic, endophytic and rhizoplanic bacterial communities of three Indian cultivated rice and one wild rice genotypes. Based on the results, carboxylic acids were preferred by microbime of *Oryza sativa* var. Sabita. Moreover, research on bacterial communities on leaves of eight perennial species naturally occurring in a Mediterranean ecosystem showed that carbohydrates were consistently used by all communities, whereas the use of carboxylic acids varied among the species [56]. The current results partially agree with the other studies. Differences in the metabolism of different groups of compounds are probably due to the predominance of different bacterial groups on individual leaves, which in turn is influenced by leaf structure and chemical composition, as well as by the abiotic factors [61]. 

Only a few studies on the metabolic profile of the plant microbiome based on the Biolog EcoPlate assay are available, and hence these results can expand the knowledge in this area [32,40,57,60]. This study followed the standard Biolog method proposed by the manufacturer’s instructions to have the opportunity to compare the obtained results with other exterminates. However, it should be noted that experimentally different incubation temperatures might be a useful way to see how community function changes over the day/season. Many researchers indicate that temperature is an important environmental factor that can greatly influence the metabolic activity as well as the composition and diversity of the microbial community in different samples [62,63,64]. 

### 3.2. Microbial Structures 

**Figure 6:**
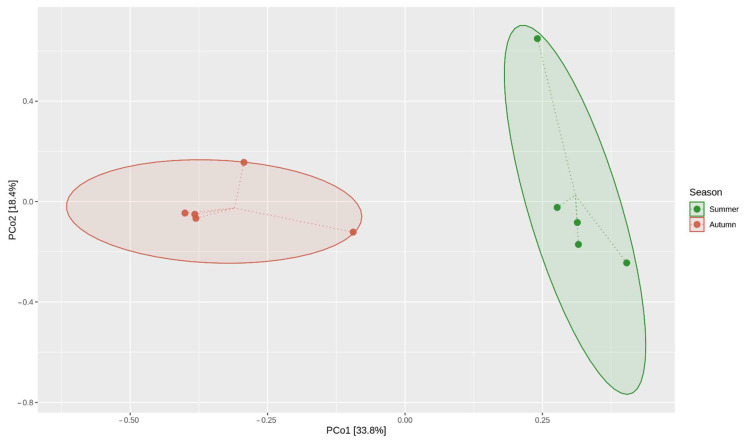
Principal coordinate analysis (PCoA) based on the overall structure of the endosphere microbiota in all samples. The data used for the PCA analysis are data derived from metagenomic studies (16S rDNA amplicon sequencing). Each data point represents an individual sample. PCoA was calculated using Bray–Curtis distances with a multivariate t-distribution. Ellipses represent a 90% confidence level. Colour is indicative of cohort.

In the current research, the bacterial communities in the *Paulownia* endosphere were dominated by Proteobacteria and Actinobacteria. Our earlier studies on *Paulownia* clones found that different tissues, including leaves, were colonised by a diverse array of endophyte communities [32]. Other studies have shown that in the endosphere of healthy leaves and leaves with symptoms of chlorosis and necrosis, Proteobacteria, Bacteroidetes and Actinobacteria dominated [37]. The shifts in community composition in the *Paulownia* leaves between young trees [32] and older trees [37], associated with the dominance of Bacteroidetes on young leaves, may be largely related to the differences in leaf age, and thus, specific leaf characteristics that include leaf chemical composition and cuticle structure and composition [65]. In studies analysing bacteria in the endosphere of a number of plants, Proteobacteria are considered the core dominant phyla, accounting for approximately 50 % of the total bacteria, which is consistent with our results [65,66]. A high relative abundance of this phylum has been reported for different tree species, including apple [67], ash [68] and eucalyptus [50]. The bacterial communities inhabiting the leaves of a given hybrids varied within and across individuals from different localities; however, there was far more variability in the bacterial community structure across the different seasons (Figure 6).

The microbiota associated with the plant host is shaped by a wide range of environmental factors, including geographic locality, plant phenotype and genotype, soil type, as well as seasonal effects. This metagenomics study showed that the *Paulownia* endophytic bacterial communities are highly dynamic. Principal coordinate analysis (β-diversity analysis) indicated that the season was the main determinant of the endophyte community structure associated with *Paulownia* leaves (Figure 6). These observations emphasise the key role of seasonal effects on shaping the bacterial community of endophytes, which has also been reported for elm [69], urban trees [70], and grape [71]. In these studies, endophytic colonisation increased during the rainy and warm periods, which agrees with the findings of our study. The temporal variations in the endophytic microbiome could be explained by changes in the optimal temperature for microbial growth, as well as the physiology of the leaves [72]. Wang et al. [61] reported that two mechanisms are mainly responsible for changes in the microbial community structure. The first one is related to changes in seasonal abiotic conditions and the second one is connected with changes in leaf chemistry and photosynthetic products. 

Spatial variability was also observed in our study. Although a single hybrid was analysed, the structure of the leaf endophyte communities differed among the localities. Nevertheless, among the identified bacterial AVS, nine bacterial genera among others *Methylobacterium*, *Klenkia*, *Pseudomonas*, *Massilia*, *Cutibacterium* and *Kineococcus* were consistently found in *Paulownia* leaves from all experimental sites. These endophyte communities may be part of the core endomicrobiome of these *Paulownia* genotypes. It should be emphasised that probably the core-microbiome will remain unchanged in spatio-temporal scales, but the structure of the residual microbiome is highly unstable [73]. The results from our previous study of *Paulownia* leaf communities provide some insights into the structure of the microbiome, indicating the presence of bacteria of the genus *Methylobaterium* and *Kineococcus* [37]. The bacteria composing the endophytic bacterial core microbiome identified in this study are, among others, *Methylobaterium*, *Psudomonas* and *Kineococcus*, which have also been found in the endosphere of mulberry trees [74], *Methylobaterium* and *Massilia,* which have also been identified in tea leaves [75]. 

The obtained research results can enrich the existing knowledge of endophytic bacteria, their interactions with plants, their diversity and mechanisms of promoting plant growth and development. The research can provide a basis for developing innovative strategies for plant adaptation to climate change and reducing biodiversity loss by creating selected bioproducts with bacterial endophytes as the main component. Plant-associated bacterial endophytes show a promising potential in increasing the productivity of agricultural crops exposed to environmental stress and nutrient deficiencies. The use of plant-growth-promoting bacterial endophytes in agriculture has a great potential to reduce the negative environmental impact of chemical fertilisers and pesticides. Thus, in the perspective of long-term management of soil fertility and crop productivity, microbial-based fertilisers are proving to be an integral and necessary component of sustainable agriculture. The use of live microorganisms (endophytes) or their metabolites in agricultural practice is in line with the latest tenets of sustainable agriculture, especially integrated pest management (IPM) and organic farming [17,76]. 

## 4. Materials and Methods

### 4.1. Microbial Community Structure Analysis

The tree species investigated in this work was *Paulownia elongata* × *fortunei.* The leaf samples were collected in summer (5 June) and autumn (18 September) of 2017 from five plantations located in Poland: Barciany (Warmian-Masurian Voivodeship); Granice (Lublin Voivodeship); Gubin (Lubusz Voivodeship); Otrebusy (Masovian Voivodeship); and Podkampinos (Masovian Voivodeship). 

The leaves were cut off from each tree, immediately placed into a sterile bag plastic bag, and transported on a piece of ice to the laboratory, where they were kept at 4 °C before being processed (within 1 days). Only healthy fresh leaves of 1-year-old trees of *Paulownia* spp. were selected. To eliminate the interference of other factors, each leaf was washed and rinsed with distilled water to remove attached clay and other microorganisms. The process of surface sterilisation was as follows: pieces were immersed in 70% ethanol, 2% sodium hypochlorite (NaClO), and 70% ethanol for 30 s, and followed by three washes with sterile distilled water and 0.9% NaCl. The effectiveness of this disinfection technique was confirmed by inoculating 100 µL of the final rinse water onto Tryptic Soy Agar (TSA) (Sigma-Aldrich, St. Louis, MO, USA), which consistently yielded no growth of bacterial and fungal colonies. Five grams of each leaf sample was ground with an aqueous solution (5 mL 0.9% NaCl), using a sterile mortar and pestle [32,60,77,78]. After sterilisation, part of the liquid sample was reserved for the analysis of the microbiome structure, and the rest was used to analyse the metabolic profile of three biological replicates for each leaf. Samples were labelled according to the collection site. The sample abbreviations, and number of samples examined by current research are shown in Table 2.

### 4.2. Microbial Community Analysis Using Biolog EcoPlates^TM^

The functional diversity of the microbial communities in the from all leaf samples was determined using Biolog EcoPlates (Biolog, Inc., Hayward, CA, USA). BIOLOG EcoPlates^TM^ is a phenotype microarray technique that allows determining the metabolic activity of heterotrophic microbial populations by analysing the characteristic usage pattern of carbon sources at defined time intervals. The use of 31 different carbon sources by the microorganisms allows to generate a physiological profile (community-level physiological profiles—CLPP), referred to as the metabolic/phenotypic fingerprinting or metabolic functions [41,42,43,79].

Analysis of the functional diversity of microbial communities was carried out following a protocol modified by us. The liquid materials obtained after maceration were used for the preparation of serial dilutions. For inoculation of the Biolog EcoPlates 10^−3^, serial dilutions were used. Samples were shaken (30 min, 140 rpm, 20 °C), and the extracts were kept at 4 °C for 30 min. Each well on a plate was inoculated with 120 μL of the previously filtered microbial suspension from leaf extracts. Afterwards, the plates were cultured at 25 °C in an incubator for 7 days. Finally, absorbance at 590 nm (optical density) in the wells was measured at 24 h intervals using a MicroStation ID system by Biolog (Biolog Inc., Hayward, CA, USA) [32,40,56,60,77]. The microbial activity in each sample was expressed as the average well-colour development (AWCD). The AWCD was calculated according to the equation AWCD = ∑ODi/31, where ODi represents the absorbance value of the control wells, corrected subtracting the blank well (inoculated, but without a carbon source). In addition, functional diversity (Shannon diversity H′, Richness S and Shannon evenness E indices) was assessed from the Biolog EcoPlate results. The Shannon–Weaver index was calculated as follows: H = −∑pi(lnpi). Here, pi is the ratio of the activity on each substrate (ODi) to the sum of activities on all substrates ∑ODi. Richness (R) values were calculated as the number of oxidised C substrates, using an OD of 0.25 as threshold for positive response. Shannon’s evenness index (E), which particularly focuses on the evenness of ci values across all utilised substrates E = H′ ln R. These indices were determined using the absorbance measurements from the works of Frąc et al., 2012 [41]; Sofo and Ricciuti, 2019 [59]; and Oszust and Frąc, 2021 [79]. The above-mentioned ecological indices calculated based on Biolog^®^ results should be regarded as substrates/carbon sources richness, evenness or diversity utilised by microbial community [41,59,79]. Statistical analyses were performed using the Statistica 13.1 software (StatSoft, Inc., Tulsa, OK, USA). Data were analysed by post-hoc analysis using Tukey’s honestly significant difference (HSD) test at a significance level of *p* < 0.05.

### 4.3. DNA Extraction, PCR and Illumina Amplicon Sequencing 

Total DNA for metagenomic analyses was extracted from the diluted samples (10^−3^) using the Fast DNATM SPIN Kit (MP Biomedicals, Solon, OH, USA) according to the manufacturer’s instructions. The extracted genomic DNA was quantified and checked for quality at A260/280 nm (1.7–2.0) using a NanoDrop 1000 Spectrophotometer (Thermo Fisher Scientific, Waltham, MA, USA) and diluted in sterile water to 10 ng μL^−1^. Next-generation sequencing was performed by Genomed S.A. (Warsaw, Poland) on a MiSeq sequencer (Illumina, San Diego, CA, USA) in paired-end (PE) technology, 2 × 300 nt, using the Illumina v 3 kit (San Diego, CA, USA). Meta-barcoding analysis of the microbial community was performed based on the variable regions V5–V7 of the 16S rRNA gene. Bacterial 16S rRNA gene amplification was performed using the primers 799F (AACMGGATTAGATACCCKG) [80,81] and 1193R (ACGCATCCCCACCTTCCTC) [81,82], which minimise chloroplast and mitochondria contamination. Amplification and library preparation were performed using a Q5 Hot Start High—Fidelity 2X Master Mix (New England Biolabs Inc., Ipswich, MA, USA), according to the manufacturer’s instructions.

### 4.4. Bioinformatic Analysis

Amplicon sequence variants (ASVs) were obtained with the DADA2 v.1.8 package [83] in R v.3.4.3 [84] (parameters: filterAndTrim—the forward and reverse sequences were trimmed to 250 bp, the first-left 20 bp were removed, maxN = 0, maxEE = 5, truncQ = 2). Chimeric sequences were removed by RemoveBimeraDenovo. Naïve Bayesian Classifier [85] (minBoot parameter = 50) was used for taxonomy assignment against the latest version of the RDP database. Non-bacterial (mitochondrial and chloroplasts) sequences were filtered out (subset_taxa function in the phyloseq package) [86]. The PCoA analysis and graphs for taxonomy abundance were prepared in R v.3.4.3 using the microeco package (v.0.7.1) [87].

## 5. Conclusions

There is a growing interest in biological methods of increasing the yield and quality of plant crops, caused both by the increased interest in sustainable and ecological agriculture, and economic benefits. Across Europe, in recent years, various efforts have been made to increase the acreage of energy crops. In conventional agriculture, nitrogen deficiencies are supplemented chemically through the use of synthetic fertilisers. However, this method is energy intensive, expensive and has a negative impact on the environment. The use of biotechnologically important bacterial endophytes as constituents of fertilisers could increase the yield potential of these plants. Our preliminary studies show that *Paulownia elongata* × *fortunei* has a rich composition of endosphere microbiota associated with the leaves of bioenergy tree. Hence, further studies will be based on the selection and detailed characterisation of these important bacteria. Additionally, our research is one of the few studies covering such complex structural and metabolic characteristics of leaf endophytes.

## Figures and Tables

**Table 1:** Comparison of metabolic functional diversity indices of the microbial communities in leaf samples based on substrate utilisation patterns on Biolog EcoPlates containing 31 different carbon sources. Different letters indicate significant differences (*p* < 0.05, *n* = 3) by Tukey’s HSD test.

No.	Location	Sample	AWCD	Richness (R)	Shannon Diversity (H’)	Shannon Evenness (E)
1.	Barciany	I_BS	0.592 ± 0.066 ^d^	13.000 ± 4.000 ^d^	2.634 ± 0.225 ^cd^	0.788 ± 0.071 ^bc^
2.		II_BA	1.047 ± 0.046 ^ab^	24.667 ± 1.155 ^ab^	3.175 ± 0.025 ^a^	0.937 ± 0.012 ^a^
3.	Granice	I_GS	0.448 ± 0.097 ^d^	12.333 ± 1.528 ^d^	2.529 ± 0.113 ^d^	0.790 ± 0.075 ^bc^
4.		II_GA	1.179 ± 0.042 ^a^	27.333 ± 2.082 ^a^	3.218 ± 0.051 ^a^	0.962 ± 0.007 ^a^
5.	Gubin	I_GuS	1.007 ± 0.064 ^ab^	17.667 ± 0.577 ^cd^	2.823 ± 0.047 ^bc^	0.867 ± 0.064 ^abc^
6.		II_GuA	0.835 ± 0.027 ^c^	20.333 ± 1.155 ^bc^	3.038 ± 0.02 ^ab^	0.913 ± 0.025 ^ab^
7.	Otrebusy	I_OS	0.909 ± 0.108 ^c^	24.667 ± 1.528 ^ab^	3.134 ± 0.041 ^a^	0.928 ± 0.02 ^a^
8.		II_OA	0.927 ± 0.109 ^c^	25.000 ± 1.732 ^ab^	3.148 ± 0.033 ^a^	0.929 ± 0.014 ^a^
9.	Podkampinos	I_PS	0.414 ± 0.057 ^d^	14.333 ± 3.215 ^cd^	2.663 ± 0.032 ^cd^	0.860 ± 0.043 ^abc^
10.		II_PA	1.215 ± 0.074 ^a^	24.667 ± 2.517 ^ab^	3.160 ± 0.079 ^a^	0.939 ± 0.012 ^a^

**Table 2:** Leaf samples labelling.

No.	Location	Sampling Season	Abbreviations
1.	Barciany	Summer	I_BS
2.		Autumn	II_BA
3.	Granice	Summer	I_GS
4.		Autumn	II_GA
5.	Gubin	Summer	I_GuS
6.		Autumn	II_GuA
7.	Otrebusy	Summer	I_OS
8.		Autumn	II_OA
9.	Podkampinos	Summer	I_PS
10.		Autumn	II_PA

## Data Availability

The raw data presented in this study are available on request from the corresponding author. The data are not publicly available due to intellectual property.

## Abbreviations

AWCD	The Average Colour development
AVS	Amplicon Sequence Variants
CLPP	Community Level Physiological Profiling
E	Shannon evenness index
H’	Shannon diversity index
HSD test	Honestly Significant Difference test
IPM	Integrated Pest Management
PCoA	Principal coordinate analysis
R	Richness index
RDP	Ribosomal Database Project
TSA	Tryptic Soy Agar
